# Effects of Ischemic Acute Kidney Injury on Lung Water Balance: Nephrogenic Pulmonary Edema?

**DOI:** 10.1155/2011/414253

**Published:** 2011-05-24

**Authors:** Rajit K. Basu, Derek Wheeler

**Affiliations:** ^1^Division of Critical Care Medicine, Cincinnati Children's Hospital Medical Center, 3333 Burnet Avenue, Cincinnati, OH 45229, USA; ^2^Cincinnati Children's Hospital Medical Center, 3333 Burnet Avenue, Cincinnati, OH 45229, USA

## Abstract

Pulmonary edema worsens the morbidity and increases the mortality of critically ill patients. Mechanistically, edema formation in the lung is a result of net flow across the alveolar capillary membrane, dependent on the relationship of hydrostatic and oncotic pressures. Traditionally, the contribution of acute kidney injury (AKI) to the formation of pulmonary edema has been attributed to bulk fluid accumulation, increasing capillary hydrostatic pressure and the gradient favoring net flow into the alveolar spaces. Recent research has revealed more subtle, and distant, effects of AKI. In this review we discuss the concept of nephrogenic pulmonary edema. Pro-inflammatory gene upregulation, chemokine over-expression, altered biochemical channel function, and apoptotic dysregulation manifest in the lung are now understood as “extra-renal” and pulmonary effects of AKI. AKI should be counted as a disease process that alters the endothelial integrity of the alveolar capillary barrier and has the potential to overpower the ability of the lung to regulate fluid balance. Nephrogenic pulmonary edema, therefore, is the net effect of fluid accumulation in the lung as a result of both the macroscopic and microscopic effects of AKI.

## 1. Introduction

Pulmonary edema, a state of abnormal fluid accumulation in the alveolar space of the lung, can interfere with normal oxygenation and ventilation. At baseline, the heart, lungs, and vascular system work in concert to facilitate fluid movement through the capillary and lymphatic beds of the lung ensuring adequate oxygen transport with limited transudation of fluid from the lung interstitium into the alveolar space. In disease states causing acute lung injury (ALI), both direct and indirect, this harmony can be disrupted. 

Pulmonary edema affects the morbidity and overall mortality of critically ill patients. Patients with pulmonary edema have longer hospital stays, duration of mechanical ventilation, and rates of pneumonia in both adults and children [1–3]. In adult and pediatric populations, pulmonary edema can complicate the hospital course of any patient, but has notable effects on those with primary respiratory failure, transplant surgery, cardiothoracic surgery, or traumatic brain injury. More recently, clinicians are appreciating the impact of acute kidney injury (AKI) on ventilation and water balance in the lung. As clinical and experimental evidence continues to mount for organ crosstalk, the relationship between the kidney and the lung in critical illness emerges as pathologically significant [4]. 

The purpose of this brief narrative is to discuss the potential impact of AKI on lung homeostasis, specifically water balance. Numerous laboratory models of isolated AKI have demonstrated deleterious effects on lung pathology and support the existence of kidney-lung crosstalk. Using this background, we propose the concept of nephrogenic pulmonary edema. Though fluid overload is often seen in patients with acute renal failure, we highlight the evidence pointing to a state of pulmonary edema induced by extrarenal microscopic effects of kidney injury. Timely identification of these effects of AKI may aid in the treatment of this disease process which has significant associated morbidity and mortality [5].

## 2. Pathophysiology

**Figure 1:**
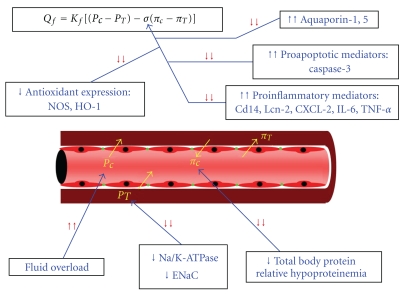
Nephrogenic pulmonary edema. Acute kidney injury triggers numerous mechanisms which alter the homeostasis of pulmonary interstitial and alveolar fluid balance. *Q*_*f*_: net flow across the alveolar-capillary membrane; *K*_*f*_: filtration coefficient; *P*_*C*_: capillary hydrostatic pressure; *P*_*T*_: interstitial fluid hydrostatic pressure; *σ*: reflection coefficient of the alveolar-capillary membrane; *π*_*C*_: capillary oncotic pressure; *π*_*T*_: interstitial oncotic pressure; NOS: nitric oxide synthase; HO-1: heme-oxygenase-1; Lcn-2: lipocalin-2; CXCL-2: chemokine ligand-2; IL-6: interleukin-6; TNF-*α*: tumor necrosis factor-*α*; Na/K-ATPase: sodium-potassium ATPase; ENaC: endothelial sodium channel. It is important to note that negative changes in the reflection coefficient likely also increase the filtration coefficient leading to a net increase in fluid flow across the alveolar-capillary membrane.

Pulmonary edema occurs when net flow across the alveolar-capillary membrane is positive. Under normal conditions, fluid in the alveolar space is drained by pulmonary lymphatic vessels and then contained within the interstitial space. If the alveolar epithelial barrier is disrupted or if the interstitial fluid volume of the lungs increases by more than 50%, flooding of the alveolar space can occur [6]. The movement of fluid is dependent on both hydrostatic and oncotic pressures and is modeled by the Starling equation

(1) $$Q_{f}=K_{f}\left[\left(P_{C}-P_{T}\right)-\sigma \left(\pi_{C}-\pi_{T}\right)\right],$$

where *Q*_*f*_ is the net flow across the alveolar-capillary membrane; *K*_*f*_ is the filtration coefficient (dependent on the permeability and the surface area of the membrane); *P*_*C*_ is the capillary hydrostatic pressure; *P*_*T*_ is the interstitial fluid hydrostatic pressure; *σ* is the reflection coefficient of the alveolar-capillary membrane (*σ* = 1 if the membrane is impermeable to protein, and *σ* = 0 if the membrane is completely permeable to protein); *π*_*C*_ is the capillary oncotic pressure; *π*_*T*_ is the interstitial oncotic pressure [7]. In normal conditions, capillary hydrostatic pressure (*P*_*C*_) approximates interstitial fluid hydrostatic pressure (*P*_*T*_), (*P*_*C*_≃*P*_*T*_), such that net fluid movement (*Q*_*f*_) is low and fluid stays in the capillary lumen. Similarly, capillary oncotic pressure (*π*_*C*_) is normally greater than interstitial oncotic pressure (*π*_*T*_), and since the reflection coefficient (*σ*) is generally near 1 (0.7–0.95), *Q*_*f*_ is generally low. Implicit in the equation is that any force or mechanism that creates a large difference between the hydrostatic forces and the oncotic forces will increase *Q*_*f*_, and thus flux of fluid from the capillary bed into the alveolar space. The lung has safety measures in place to prevent such leakage. The lymphatic system has high capacitance and flow to clear excess fluid from the interstitium. The capillary endothelium between the interstitium and the alveolar beds can tolerate increasing amounts of hydrostatic and interstitial fluid pressure [8]. However, in conditions of excess lung water such as pulmonary edema, overall oncotic pressure becomes dilute (thereby increasing the discrepancy between the hydrostatic and the oncotic pressures) favoring net flow of fluid into the alveolar space (Figure 1). 

Aside from bulk fluid and protein effects, changes at the microscopic level change permeability of the alveolar capillary endothelium. Decreased expressions of sodium transport molecules, such as the epithelial sodium channel (ENaC), the sodium-potassium-ATPase pump (Na^+^/K^+^-ATPase), or the cystic fibrosis transmembrane conductance regulator (CFTR), all contribute to increased alveolar fluid accumulation in experimental models of pulmonary edema [9, 10]. ENaC promotes sodium absorption from the alveolar space into the lung epithelial cells. Similarly, the sodium ATPase pump allows for sodium extrusion from the alveolus. Aberrant expression of either has significant ramifications for fluid balance as water passively follows sodium. Additionally, the regulation of fluid clearance by endogenous catecholamines, particularly the *β*-adrenergic system, has been highlighted [11]. 

Variations in vascular preload, capillary endothelial integrity, and aberrations in osmotic pressure can alter the interstitial-alveolar fluid balance. Increased vascular preload leading to pulmonary edema is commonly observed in patients with left-sided cardiogenic shock and disorders of incomplete pulmonary vascular drainage. In these situations, the capacitance of the interstitial lymphatic system is overwhelmed, the *P*_*C*_ ≫ *P*_*T*_, and fluid spills from the vascular space down a hydrostatic gradient. Acutely negative intrathoracic pressure raises left ventricular stroke work and likely leads to backup of fluid into the pulmonary bed (increasing *P*_*C*_) in postobstructive pulmonary edema. The integrity of the alveolar capillary endothelium is altered in acute respiratory distress syndrome (ARDS), sepsis, and stress states [12, 13]. Bacterial contribution to type II pneumocyte surfactant production has been linked to increased capillary permeability in sepsis [14]. Such disease states alter the reflection coefficient (*σ*), decreasing the ability of oncotic pressure to balance hydrostatic pressure in the Starling equation. Neurogenic pulmonary edema, commonly observed in intracranial hypertension, may both alter the sympathetic catecholamine regulation of the systemic circulation, thereby diverting blood to the alveolar capillary bed (↑*P*_*C*_), and disrupt capillary integrity [15]. Finally, acute or chronic disease states commonly carry the comorbidity of hypoproteinemia. Low circulating serum total protein levels directly reduce the capillary oncotic pressure, favoring fluid efflux from the luminal space. The interstitial-alveolar interface in the lung is under tight control and can be affected by disease states that cause dysregulation in total body fluid status, levels of inflammatory mediators, and aberrant protein homeostasis.

## 3. AKI and Fluid Balance

Acute kidney injury complicates critical illness in many ways. Concomitant with the diagnosis of AKI was fluid overload, which has been found retrospectively to independently increase morbidities such as duration of mechanical ventilation and hospital length of stay, and overall mortality [16–18]. Fluid overload carries increased risk of morbidity in mortality in both adults and children [19]. On retrospective review, children with greater degrees of fluid overload had higher mortality on initiation of renal replacement therapy [20].

The kidney is central to many homeostatic mechanisms in the body, and as AKI progresses dysregulation occurs in various locations in the body. The kidney communicates with the lung in several ways: regulating acid-base balance, increasing oxygen carrying capacity through erythropoiesis, and regulating blood pressure through the renin-angiotensin-aldosterone axis. All of these processes may be altered in AKI. The deleterious connection between the kidney and the lung in disease states, first reported by Bass and Singer in 1950, was assumed to be secondary to uremia and fluid overload [21]. However, despite the use of dialysis to limit uremia and to control fluid balance, high mortality rates continue in patients with both AKI and ALI [22]. In progressive ALI and ARDS, impaired global oxygenation will necessarily decrease renal oxygenation leading to a vicious cycle of progressive lung and kidney injury [23]. The crosstalk between the kidney and the lung in the critically ill patient is of extreme importance to all critical care physicians [4].

## 4. AKI and ALI

The impact of AKI on the lung can be seen on many levels. One of the major effects of AKI is to effect lung water balance. While fluid overload from anuric or oliguric AKI can upset the hydrostatic-oncotic balance in the pulmonary interstitium, inflammation and endothelial injury which may be triggered by AKI also upset this equilibrium. The contributions of the latter, inflammation, changes in expression of regulators of the pulmonary fluid and electrolyte chemical gradient, and altered regulation of oxidative stress, may be greater than previously appreciated.

AKI triggers gene expression changes which may alter lung vascular stability. Global gene expression mapping of lung tissues in experimental murine kidney ischemia identifies proinflammatory and proapoptotic gene upregulation in the lung transcriptome [24]. Proinflammatory genes such as Cd14, lipocalin-2, chemokine ligand-2 (CXCL2), and IL-6 are all upregulated after ischemia [24]. These mediators, notably IL-6, trigger acute phase responses to antigens and initiate the inflammatory cascade. Subsequent injury is caused by many mechanisms including: disruption of endothelial integrity, aberrant signaling of the coagulation and contact cascades, and direct cellular toxicity in vital organs. Reduction of the IL-6 effect using chemical inhibition of IL-6 or use of IL-6-deficient mice reduces lung inflammation after ischemic AKI [25]. Caspase-3, a marker of cellular apoptotic activity, is upregulated in type II pneumocytes after experimental AKI [26]. Macrophages, mediators of global injury and repair, may be involved; use of macrophage activation inhibitor decreases the amount of pulmonary capillary leakage in murine ischemia [27].Uremia may contribute to such upregulation, as lung inflammation and apoptosis are abrogated after nephrectomy [28]. In repeated animal models of AKI, proinflammatory mediators are expressed within hours of injury. Circulating inflammatory cytokines may affect lung capillary endothelium, changing permeability. Apoptosis of either capillary endothelial cells or pneumocytes responsible for producing surfactant will also change the permeability of the interstitial-alveolar interface in the lung. Cytokine, chemokine, and apoptotic dysregulation is therefore a prime suspect of ALI after AKI and demonstrates kidney-lung crosstalk.

Fluid and electrolyte conduction in the lung is altered in AKI. Rabb and colleagues published the seminal work describing the downregulation of pulmonary ENaC, Na/K ATPase, and aquaporin-5 after ischemic injury and nephrectomy in rats [29]. As the primary interstitial solute, sodium is the central determinant of fluid shift based on osmolar gradients. Changes in sodium balance on either side of the capillary membrane, therefore, may have marked effects on fluid shifts between the interstitium and the alveolar bed. Aquaporin-1 message upregulation, in a sheep model of cardiopulmonary bypass and hypothermic circulatory arrest, was correlated with increased rates of pulmonary edema [30]. Similarly, aquaporin-5 has been identified in sepsis as a contributor to dysregulation in tissue and vascular permeability [31]. In experimental models, osmotically driven water permeability between the alveolar and capillary components of the lung is changed tenfold by deletion of aquaporin-1 or aquaporin-5 [32]. While changes in aquaporin expression may have only minor clinical ramification, the evidence for kidney-lung crosstalk is robust [33]. Aberrant salt and water handling by the lung is altered in even mild models of experimental kidney ischemia. The impact of this on pulmonary edema formation is obvious.

Oxidative stress plays a role in the kidney-lung crosstalk seen during AKI. Hepatic levels of superoxide dismutase, glutathione, and catalase are decreased after ischemic AKI in mice, which may decrease host response to oxidative stress [34]. Additionally, TNF-*α* levels were significantly higher in ischemic mice than in sham controls pointing to a systemic oxidative/inflammatory response of AKI [35]. The nitric oxide signaling pathway is critical to oxidative balance and is altered in AKI. Patients with chronic kidney disease have lower basal production of nitric oxide compared to controls, and mice subjected to subtotal nephrectomy have lower levels of nitric oxide synthase [36, 37]. Finally, mice lacking heme-oxygenase-1 (HO-1), critical for reducing oxidative stress and generation of antioxidant metabolites, have increased levels of inflammation after ischemic AKI versus sham controls [38]. Nitric oxide, HO-1, and TNF-*α* all affect lung capillary stability [39–41]. Therefore, the alteration in these regulators of oxidative balance after AKI can affect lung vascular stability. 

The conclusion that significant lung pathology results from ischemic AKI models must be tempered by the fact that, in practice, renal hypoperfusion without concurrent pulmonary hypoperfusion is rare. Indeed, it is rarely known if ischemia to the kidney occurs before or after ischemia to the lungs. Models of hemorrhagic and septic shock do not adequately address this issue. Additionally, in the common case of decreased renal preload (i.e., prerenal azotemia), the microscopic effects on the lung have not been clearly described. Still, the findings described above highlight the potential contribution of the injured renal endothelium to lung injury and edema formation.

## 5. Nephrogenic Pulmonary Edema

In the context of ischemic AKI and kidney-lung crosstalk, we propose the concept of *nephrogenic pulmonary edema. *Resting on the evidence of experimental extrarenal effects of AKI and the acknowledged clinical effects of oliguric volume overload, nephrogenic pulmonary edema (NPE) could represent net aberrant fluid handling at the interstitial-alveolar lung interface. On a microscopic level, NPE could result as a consequence of damage of pulmonary endothelium by AKI-mediated upregulated inflammation, disruption of pulmonary sodium transport, and activation of apoptosis in pulmonary cells responsible for maintaining the homeostatic integrity of lung water balance. AKI should be counted amongst the numerous disease processes that alter both the volume load in the pulmonary capillary bed and the endothelial integrity of the alveolar capillary barrier.

Experimental manifestations of kidney injury are manifest in multiple extrarenal locations [42]. Experimental AKI has deleterious effects on the central nervous system affecting glial cell viability, blood-brain barrier permeability, and neurocognitive status [43]. Systolic ventricular function is negatively affected by ischemic kidney injury [44]. Leukocyte trafficking is aberrant, and a host of proinflammatory genomic and proteomic responses occur after experimental AKI [24, 45]. The experimental evidence for lung injury after AKI is robust [4]. 

Critically ill patients with acute lung injury with AKI have worse morbidity and mortality. Fluid overload and uremia may be regulated by extracorporeal therapy such as dialysis, but lung injury and kidney injury are progressive and mortality rates in patients with both disease processes have not significantly improved with these therapies. Use of stratification criteria has retrospectively demonstrated improved outcomes for patients with less severe AKI [46]; biomarker research seeks to identify kidney injury in its early stages. Appreciation of the impact of kidney injury during this epoch, before the onset of oliguria and fluid overload, may be critical. The impact of kidney-lung crosstalk on the molecular level may be creating a milieu of lung inflammation, mediated by proinflammatory, oxidative, chemical, and apoptotic signals. Ultimately, the accumulated signals overpower the ability of the lung to regulate fluid balance, leading to nephrogenic pulmonary edema.

## References

[1] Kendirli T, Kavaz A, Yalaki Z, Öztürk Hişmi B, Derelli E, Ince E (2006). Mechanical ventilation in children. *Turkish Journal of Pediatrics*.

[2] Vasudevan A, Lodha R, Kabra SK (2004). Acute lung injury and acute respiratory distress syndrome. *Indian Journal of Pediatrics*.

[3] Schuller D, Mitchell JP, Calandrino FS, Schuster DP (1991). Fluid balance during pulmonary edema; is fluid gain a marker or a cause of poor outcome?. *Chest*.

[4] Ko GJ, Rabb H, Hassoun HT (2009). Kidney-lung crosstalk in the critically ill patient. *Blood Purification*.

[5] Hoste EA, Schurgers M (2008). Epidemiology of acute kidney injury: how big is the problem?. *Critical Care Medicine*.

[6] Guyton AC, Hall JE (1996). *Textbook of Medical Physiology*.

[7] Chase MP, Wheeler DS, Wheeler DS, Wong HR, Shanley TP (2007). Pediatric critical care medicine. *Basic Science and Clinical Evidence*.

[8] Demling RH, LaLonde C, Ikegami K (1993). Pulmonary edema: pathophysiology, methods of measurement, and clinical importance in acute respiratory failure. *New Horizons*.

[9] Berthiaume Y, Folkesson HG, Matthay MA (2002). Lung edema clearance: 20 Years of progress invited review: alveolar edema fluid clearance in the injured lung. *Journal of Applied Physiology*.

[10] Verghese GM, Ware LB, Matthay BA, Matthay MA (1999). Alveolar epithelial fluid transport and the resolution of clinically severe hydrostatic pulmonary edema. *Journal of Applied Physiology*.

[11] Mehta D, Bhattacharya J, Matthay MA, Malik AB (2004). Integrated control of lung fluid balance. *American Journal of Physiology*.

[12] Berkowitz DM, Danai PA, Eaton S, Moss M, Martin GS (2008). Accurate characterization of extravascular lung water in acute respiratory distress syndrome. *Critical Care Medicine*.

[13] Groeneveld AB (2002). Vascular pharmacology of acute lung injury and acute respiratory distress syndrome. *Vascular Pharmacology*.

[14] Oldham KT, Guice KS, Stetson PS, Wolfe RR (1989). Bacteremia-induced suppression of alveolar surfactant production. *Journal of Surgical Research*.

[15] Chen HI (2009). From neurogenic pulmonary edema to fat embolism syndrome: a brief review of experimental and clinical investigations of acute lung injury and acute respiratory distress syndrome. *Chinese Journal of Physiology*.

[16] Hoste EA, De Corte W (2007). Epidemiology of AKI in the ICU. *Acta Clinica Belgica*.

[17] Hoste EAJ, Kellum JA (2007). Incidence, classification, and outcomes of acute kidney injury. *Contributions to Nephrology*.

[18] Thadhani R, Pascual M, Bonventre JV (1996). Acute renal failure. *The New England Journal of Medicine*.

[19] Bagshaw SM, Cruz DN (2010). Fluid overload as a biomarker of heart failure and acute kidney injury. *Contributions to Nephrology*.

[20] Basu RK (2010). *An Update and Review of Acute Kidney Injury in Pediatrics*.

[21] Bass HE, Singer E (1950). Pulmonary changes in uremia. *Journal of the American Medical Association*.

[22] Mehta RL, Pascual MT, Gruta CG, Zhuang S, Chertow GM (2002). Refining predictive models in critically ill patients with acute renal failure. *Journal of the American Society of Nephrology*.

[23] Kuiper JW, Groeneveld ABJ, Slutsky AS, Plötz FB (2005). Mechanical ventilation and acute renal failure. *Critical Care Medicine*.

[24] Grigoryev DN, Liu M, Hassoun HT, Cheadle C, Barnes KC, Rabb H (2008). The local and systemic inflammatory transcriptome after acute kidney injury. *Journal of the American Society of Nephrology*.

[25] Klein CL, Hoke TS, Fang WF, Altmann CJ, Douglas IS, Faubel S (2008). Interleukin-6 mediates lung injury following ischemic acute kidney injury or bilateral nephrectomy. *Kidney International*.

[26] Jang HR, Rabb H (2009). The innate immune response in ischemic acute kidney injury. *Clinical Immunology*.

[27] Kramer AA, Postler G, Salhab KF, Mendez C, Carey LC, Rabb H (1999). Renal ischemia/reperfusion leads to macrophage-mediated increase in pulmonary vascular permeability. *Kidney International*.

[28] Hoke TS, Douglas IS, Klein CL (2007). Acute renal failure after bilateral nephrectomy is associated with cytokine-mediated pulmonary injury. *Journal of the American Society of Nephrology*.

[29] Rabb H, Wang Z, Nemoto T, Hotchkiss J, Yokota N, Soleimani M (2003). Acute renal failure leads to dysregulation of lung salt and water channels. *Kidney International*.

[30] Tabbutt S, Nelson DP, Tsai N (1997). Induction of aquaporin-1 mRNA following cardiopulmonary bypass and reperfusion. *Molecular Medicine*.

[31] Arul M, Chinnaiyan MA, Huber-Lang M (2001). Molecular signatures of sepsis: multiorgan gene expression profiles of systemic inflammation. *American Journal of Pathology*.

[32] Verkman AS, Matthay MA, Song Y (2000). Aquaporin water channels and lung physiology. *American Journal of Physiology*.

[33] Song Y, Fukuda N, Bai C, Ma T, Matthay MA, Verkman AS (2000). Role of aquaporins in alveolar fluid clearance in neonatal and adult lung, and in oedema formation following acute lung injury: studies in transgenic aquaporin null mice. *Journal of Physiology*.

[34] Serteser M, Koken T, Kahraman A, Yilmaz K, Akbulut G, Dilek ON (2002). Changes in hepatic TNF-alpha levels, antioxidant status, and oxidation products after renal ischemia/reperfusion injury in mice. *Journal of Surgical Research*.

[35] Akbulut G, Dilek ON, Kahraman A, Köken T, Serteser M (2005). The correlation between renal tissue oxidative stress parameters and TNF-alpha levels in an experimental model of ischemia-reperfusion injury in mice. *Ulusal Travma ve Acil Cerrahi Dergisi*.

[36] Wever R, Boer P, Hijmering M (1999). Nitric oxide production is reduced in patients with chronic renal failure. *Arteriosclerosis, Thrombosis, and Vascular Biology*.

[37] Vaziri ND, Ni Z, Oveisi F, Liang K, Pandian R (2002). Enhanced nitric oxide inactivation and protein nitration by reactive oxygen species in renal insufficiency. *Hypertension*.

[38] Tracz MJ, Juncos JP, Croatt AJ (2007). Deficiency of heme oxygenase-1 impairs renal hemodynamics and exaggerates systemic inflammatory responses to renal ischemia. *Kidney International*.

[39] Jiang F, Roberts SJ, Datla SR, Dusting GJ (2006). NO modulates NADPH oxidase function via heme oxygenase-1 in human endothelial cells. *Hypertension*.

[40] Morio LA, Hooper KA, Brittingham J (2001). Tissue injury following inhalation of fine particulate matter and hydrogen peroxide is associated with altered production of inflammatory mediators and antioxidants by alveolar macrophages. *Toxicology and Applied Pharmacology*.

[41] Vigne P, Feolde E, Ladoux A, Duval D, Frelin C (1995). Contributions of NO synthase and heme oxygenase to cGMP formation by cytokine and hemin treated brain capillary endothelial cells. *Biochemical and Biophysical Research Communications*.

[42] Feltes CM, Van Eyk J, Rabb H (2008). Distant-organ changes after acute kidney injury. *Nephron Physiology*.

[43] Liu M, Liang Y, Chigurupati S (2008). Acute kidney injury leads to inflammation and functional changes in the brain. *Journal of the American Society of Nephrology*.

[44] Kelly KJ (2003). Distant effects of experimental renal ischemia/reperfusion injury. *Journal of the American Society of Nephrology*.

[45] Miyazawa S, Watanabe H, Miyaji C, Hotta O, Abo T (2002). Leukocyte accumulation and changes in extra-renal organs during renal ischemia reperfusion in mice. *Journal of Laboratory and Clinical Medicine*.

[46] Bagshaw SM, George C, Dinu I, Bellomo R (2008). A multi-centre evaluation of the RIFLE criteria for early acute kidney injury in critically ill patients. *Nephrology Dialysis Transplantation*.

